# Coronary Microvascular Dysfunction and Premature Ventricular Contractions in Patients With Stable Angina

**DOI:** 10.1001/jamanetworkopen.2025.46595

**Published:** 2025-12-03

**Authors:** Ann-Kathrin Kahle, Bahram Wafaisade, Fares-Alexander Alken, Katharina Scherschel, Ernan Zhu, Nicola A. Cicco, Alexandru Gabriel Bejinariu, Florian Bönner, Malte Kelm, Mareike Cramer, Christian Meyer

**Affiliations:** 1 Division of Cardiology, Angiology, Intensive Care Medicine, EVK Düsseldorf, cNEP, Cardiac Neuro- and Electrophysiology Research Consortium, Düsseldorf, Germany; 2 Department of Cardiology, Pulmonology and Vascular Medicine, Medical Faculty and University Hospital Düsseldorf, Heinrich Heine University, Düsseldorf, Germany; 3 Institute of Neural and Sensory Physiology, cNEP, Cardiac Neuro- and Electrophysiology Research Consortium, Heinrich Heine University Düsseldorf, Medical Faculty, Düsseldorf, Germany; 4 Cardiovascular Research Institute Düsseldorf, Medical Faculty and University Hospital Düsseldorf, Heinrich Heine University, Düsseldorf, Germany; 5 Department of Cardiology, Vascular Medicine and Pulmonology, Hospital Düren, Düren, Germany

## Abstract

This case-control study examines associations of coronary microvascular dysfunction (CMD) with occurrence of premature ventricular contractions among patients with stable angina or equivalent symptoms.

## Introduction

Coronary microvascular dysfunction (CMD) is highly prevalent in patients with angina and nonobstructive coronary artery disease resulting in adverse outcomes.^1,2,3^ The underlying causes are incompletely understood. Premature ventricular contractions (PVCs) have been known for decades to predict heart failure and mortality.^4,5,6^ Here, we hypothesized that CMD is associated with PVC occurrence, reflecting a potential pathophysiological interplay.

## Methods

The case-control study complied with the Declaration of Helsinki, was approved by the institutional review board of the Medical Association North-Rhine, and followed the STROBE reporting guideline. Patients gave written informed consent.

In this study, consecutive patients with stable angina or equivalent symptoms and available Holter electrocardiograms (December 2020 to July 2024) undergoing standardized microcirculatory function assessment^1^ within 6 months were studied. In cohort 1, CMD diagnosis was performed by stress cardiac magnetic resonance imaging (Philips Healthcare) and in cohort 2, by continuous thermodilution (PressureWire X, Abbott) using a dedicated software (CoroFlow Cardiovascular System [Coroventis]) with established diagnostic criteria.^1^

PVC prevalence was compared between patients with vs without CMD. Group comparisons used Mann-Whitney *U* or χ^2^ tests. The association of CMD with PVC burden was analyzed by logistic regression with multivariable adjustment for comorbidities. A *P* value <.05 was considered statistically significant. No participants were lost to follow-up. Missing data were minimal, confined to nonessential variables, and not subject to imputation. We used GraphPad Prism 10.5.0 (GraphPad Inc) for analysis.

## Results

Among 300 patients (median [IQR] age, 70 [60-78] years; 187 male [62.3%]), 260 underwent cardiac magnetic resonance imaging (cohort 1), and 40 underwent continuous thermodilution (cohort 2). In cohort 1, CMD was diagnosed in 89 cases (34.2%). These patients exhibited a significantly higher number of PVCs in 24 hours and a greater PVC burden than those without CMD. They more frequently exceeded clinically relevant PVC thresholds of 0.5% or greater (odds ratio [OR], 3.50; 95% CI, 2.03-6.05; *P* < .001), 5.0% or greater (OR, 2.63; 95% CI, 1.30-5.32; *P* = .006), and 10.0% or greater (OR, 3.18; 95% CI, 1.25-8.09; *P* = .01) (Table and Figure). In multivariate models adjusted for comorbidities, CMD was the only independent variable associated with a PVC burden of 0.5% or greater (OR, 3.78; 95% CI, 2.21-6.74; *P* < .001), 5.0% or greater (OR, 3.23; 95% CI, 1.51-6.93; *P* = .003), and 10.0% or greater (OR, 3.81; 95% CI, 1.41-10.27; *P* = .008). Comorbidities were comparable.

**Table.** Baseline Characteristics and Holter Analysis of Patients With vs Without CMD

Variable	Patients, No. (%)		*P* value
	Without CMD	With CMD	
Cohort 1			
Participants, No.	171	89	NA
Baseline characteristics			
Obstructive CAD	66 (38.6)	36 (40.5)	.81
Prior PCI	42 (24.6)	25 (28.1)	.54
Prior CABG	8 (4.7)	6 (6.7)	.48
CMR-detected ischemia	29 (17.0)	12 (13.5)	.47
CMR-detected LGE	29 (17.0)	10 (11.2)	.22
SHD	34 (19.9)	12 (13.5)	.20
Hypertensive heart disease	23 (13.5)	10 (11.2)	.61
LVEF, median (IQR), %	58 (52-65)	60 (54-66)	.25
TAPSE, median (IQR), mm	22 (19-25)	21 (19-24)	.76
IVSd, median (IQR), mm	10 (9-12)	10 (9-12)	.87
Diastolic dysfunction	61 (35.7)	36 (40.5)	.45
β-Receptor blockers	87 (50.9)	45 (50.6)	.96
Calcium channel blockers	32 (18.7)	19 (21.4)	.61
ACE inhibitors and ARBs	77 (45.0)	44 (49.4)	.50
Diuretics	52 (30.4)	29 (32.6)	.72
ARNIs	10 (5.9)	5 (5.6)	.94
SGLT2 inhibitors	19 (11.1)	14 (15.7)	.29
Holter analysis			
Time between Holter analysis and microcirculatory function assessment, median (IQR), d	22 (5-113)	14 (1-120)	.14
No. of PVCs per 24 h, median (IQR)	93 (5-599)	635 (112-4284)	<.001
PVC burden			
Median (IQR), %	0.001 (0.000-0.300)	0.600 (0.005-4.000)	<.001
≥0.5%	40 (23.4)	46 (51.7)	<.001
≥5.0%	17 (9.9)	20 (22.5)	.006
≥10.0%	8 (4.7)	12 (13.5)	.01
Cohort 1: Subgroup			
Participants, No.	67	33	NA
Baseline characteristics			
LVEF, median (IQR), %	62 (56-67)	62 (58-67)	.71
TAPSE, median (IQR), mm	22 (20-25)	22 (19-23)	.24
IVSd, median (IQR), mm	10 (9-11)	10 (9-11)	.72
Diastolic dysfunction	16 (23.9)	11 (33.3)	.32
β-Receptor blockers	22 (32.8)	14 (42.4)	.35
Calcium channel blockers	6 (9.0)	3 (9.1)	.98
ACE inhibitors and ARBs	22 (32.8)	11 (33.3)	.96
Diuretics	7 (10.5)	6 (18.2)	.28
ARNIs	0	1 (3.0)	.15
SGLT2 inhibitors	1 (1.5)	3 (9.1)	.07
Holter analysis			
Time between Holter analysis and microcirculatory function assessment, median (IQR), d	27 (7-160)	17 (1-86)	.15
No. of PVCs per 24 h, median (IQR)	33 (4-468)	608 (88-4024)	<.001
PVC burden			
Median (IQR), %	0.001 (0.000-0.095)	0.500 (0.005-4.200)	<.001
≥0.5%	11 (16.4)	17 (51.5)	<.001
≥5.0%	2 (3.0)	7 (21.2)	.003
≥10.0%	2 (3.0)	5 (15.2)	.03
Cohort 2			
Participants, No.	11	29	NA
Baseline characteristics			
Obstructive CAD	6 (54.5)	20 (69.0)	.39
Prior PCI	3 (27.3)	9 (31.0)	.82
Prior CABG	0	1 (3.4)	.53
LVEF, median (IQR), %	62 (51-66)	57 (54-60)	.13
TAPSE, median (IQR), mm	22 (18-23)	21 (19-24)	.61
IVSd, median (IQR), mm	10 (9-10)	11 (9-12)	.24
Diastolic dysfunction	4 (36.4)	16 (55.2)	.29
β-Receptor blockers	6 (54.6)	14 (48.3)	.72
Calcium channel blockers	2 (18.2)	7 (24.1)	.69
ACE inhibitors and ARBs	8 (72.7)	19 (65.5)	.66
Diuretics	3 (27.3)	7 (24.1)	.84
ARNIs	0	2 (6.9)	.37
SGLT2 inhibitors	0	5 (17.2)	.14
Holter analysis			
Time between Holter analysis and microcirculatory function assessment, median (IQR), d	23 (0-66)	2 (0-59)	.82
No. of PVCs per 24 h, median (IQR)	191 (89-774)	846 (180-2415)	.03
PVC burden			
Median (IQR), %	0.090 (0.001-0.150)	0.450 (0.100-2.500)	.02
≥0.5%	1 (9.1)	14 (48.3)	.02

Abbreviations: ACE, angiotensin converting enzyme; ARB, angiotensin receptor blocker; ARNI, angiotensin receptor-neprilysin inhibitor; CABG, coronary artery bypass graft; CAD, coronary artery disease; CMD, coronary microvascular dysfunction; CMR, cardiac magnetic resonance imaging; IVSd, interventricular septum thickness at end-diastole; LGE, late gadolinium enhancement; LVEF, left ventricular ejection fraction; NA, not applicable; PCI, percutaneous coronary intervention; PVC, premature ventricular contraction; SGLT2, sodium-glucose cotransporter-2; SHD, structural heart disease; TAPSE, tricuspid annular plane systolic excursion.

**Figure.**
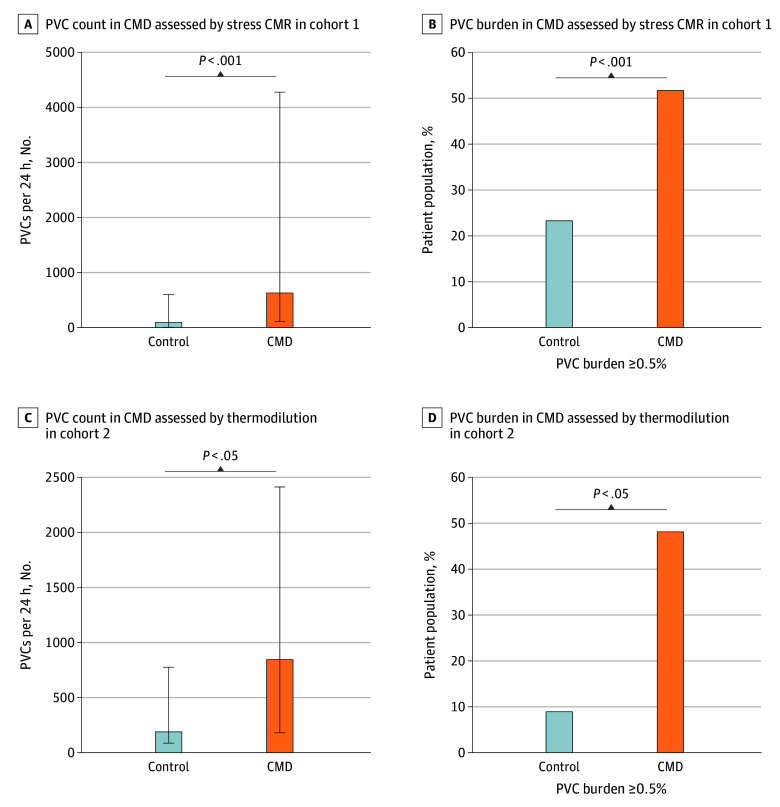
Coronary Microvascular Dysfunction (CMD) and Premature Ventricular Contraction (PVC) Occurrence Patients with CMD assessed via stress cardiac magnetic resonance imaging (CMR; A, B) and continuous thermodilution (C,D) presented with more absolute PVC counts and a more frequent PVC burden threshold of 0.5% or greater than patients without CMD. Error bars indicate interquartile ranges for median values.

In a subgroup of 100 patients without obstructive coronary artery disease, structural heart disease, ischemia, or late gadolinium enhancement, CMD was present in 33 cases (33.0%). Again, these patients had more PVCs and a higher PVC burden. CMD was associated with increased odds of exceeding PVC thresholds of 0.5% or greater (OR, 5.41; 95% CI, 2.11-13.85; *P* < .001), 5.0% or greater (OR, 8.75; 95% CI, 1.70-44.93; *P* = .003), and 10.0% or greater (OR, 5.80; 95% CI, 1.06-31.73; *P* = .03). After adjustment, CMD was the only independent variable associated with a PVC burden of 0.5% or greater (OR, 5.05; 95% CI, 1.93-13.23; *P* = .001), 5.0% or greater (OR, 9.26; 95% CI, 1.69-50.80; *P* = .01), and 10.0% or greater (OR, 6.27; 95% CI, 1.06-37.21; *P* = .045).

In cohort 2, CMD was diagnosed in 29 cases (72.5%). Again, these patients exhibited higher PVC counts and burden, with a more frequent burden of 0.5% or greater (OR, 9.33; 95% CI, 1.05-82.64; *P* = .02) (Table and Figure). CMD was the only independent variable associated with a PVC burden of 0.5% or greater after adjustment (0.5%: OR, 9.70; 95% CI, 1.04-90.91; *P* = .047). Comorbidities were comparable.

## Discussion

To our knowledge, this case-control study is the first to have found that CMD is associated with PVC occurrence; both absolute PVC counts and burden thresholds—established heart failure and mortality predictors^4,6^—were significantly increased in CMD, even after adjustment. Because this regional observational study did not address causality between CMD and ventricular ectopy, generalizability is limited. However, considering that CMD has not been reported in PVC outcome trials,^6^ the pathophysiological interplay suggested here appears to be underestimated so far, warranting future studies on targeted diagnostic and therapeutic concepts.

## Notes

Supplement. Data Sharing Statement
