# Supplementary material for: Coronary Microvascular Dysfunction and Premature Ventricular Contractions in Patients With Stable Angina
Source: JAMA Netw Open. 2025 Dec 3;8(12):e2546595. doi: 10.1001/jamanetworkopen.2025.46595 (PMC12676358; doi:10.1001/jamanetworkopen.2025.46595)
Supplement: Supplement. — Data Sharing Statement [file jamanetwopen-e2546595-s001.pdf]

Data Sharing Statement

Kahle. Coronary Microvascular Dysfunction and Premature Ventricular Contractions in Patients With Stable Angina. *JAMA Netw Open*. Published December 03, 2025.
doi:10.1001/jamanetworkopen.2025.46595

Data
Data available: Yes
Data types: Deidentified participant data, Data dictionary
How to access data: The data underlying this article will be shared on reasonable request to the corresponding author.
When available: With publication

Supporting Documents
Document types: Statistical/analytic code, Other (please specify)
Additional Information: Statistical methods
How to access documents: The data underlying this article will be shared on reasonable request to the corresponding author.
When available: With publication

Additional Information
Who can access the data: Researchers whose proposed use of the data has been approved
Types of analyses: Non-commercial analyses
Mechanisms of data availability: After approval of a proposal
